# Supplementary figures and images for: Reducing the effect of immortal time bias affects the analysis of prevention of delirium by suvorexant in critically ill patients: A retrospective cohort study
Source: PLoS One. 2022 Dec 1;17(12):e0277916. doi: 10.1371/journal.pone.0277916 (PMC9714704; doi:10.1371/journal.pone.0277916)

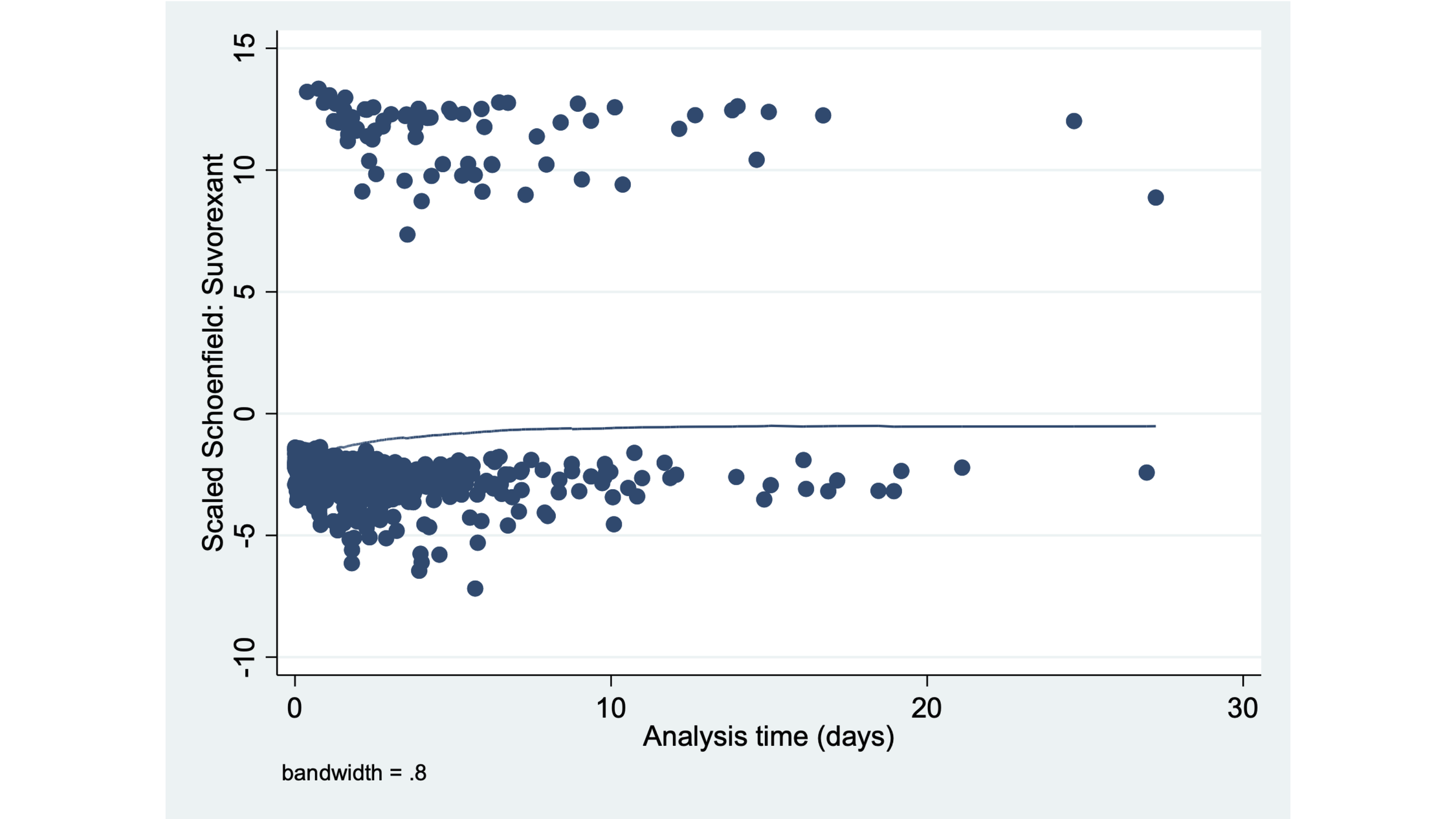

Supplement: S1 Fig — a. Schoenfield residuals of the “any time before” analysis. b. Schoenfield residuals of the “within 72h” analysis. (ZIP) [file pone.0277916.s001.zip › S1a_Fig.tif]

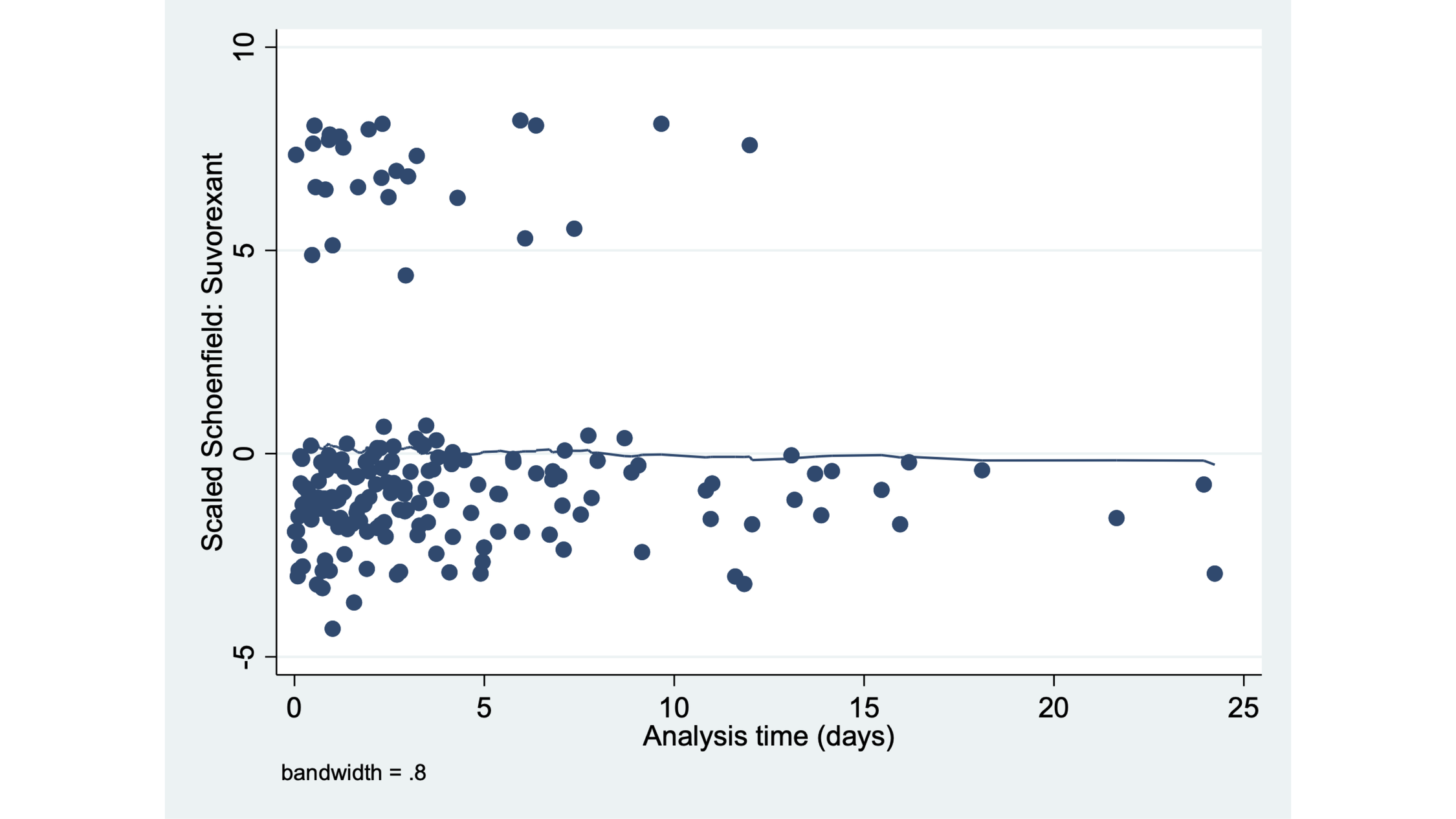

Supplement: S1 Fig — a. Schoenfield residuals of the “any time before” analysis. b. Schoenfield residuals of the “within 72h” analysis. (ZIP) [file pone.0277916.s001.zip › S1b_Fig.tif]
