# Supplementary material for: Reducing the effect of immortal time bias affects the analysis of prevention of delirium by suvorexant in critically ill patients: A retrospective cohort study
Source: PLoS One. 2022 Dec 1;17(12):e0277916. doi: 10.1371/journal.pone.0277916 (PMC9714704; doi:10.1371/journal.pone.0277916)
Supplement: S1 Table — Patients’ backgrounds in the “any time before” analysis. (DOCX) [file pone.0277916.s002.docx]

|  | Control (n=1,550) | Suvorexant (n=504) | p |
| --- | --- | --- | --- |
| Gender, Male | 999 (64.5) | 356 (70.6) | 0.01 |
| Age | 71 (62 – 78) | 70 (61 – 77) | 0.05 |
| APACHE2 | 18 (15 – 23) | 16 (13 – 20) | <0.01 |
| Maintenance HD^a^ | 115 (7.4) | 55 (10.9) | 0.01 |
| Heart failure | 15 (1.0) | 4 (0.8) | 1.00 |
| Respiratory failure | 20 (1.3) | 1 (0.2) | 0.04 |
| S/P CV^b^ surgery | 704 (45.4) | 291 (57.7) | <0.01 |
| Readmission | 102 (6.6) | 40 (7.9) | 0.30 |
| Emergency call;  RRS^c^/ code blue | 168 (10.8) / 5 (0.3) | 44 (8.7) / 0 | 0.22 |
| Admission category; non-surgery/ elective surgery/ emergent surgery | 645 (41.6)/ 523 (33.7) / 382 (24.7) | 167 (33.1)/ 263 (52.2) / 74 (14.7) | <0.01 |
| Emergency Admission | 1,025 (66.1) | 243 (48.2) | <0.01 |
| Place before the ICU; general ward/ OR^d^/ ED^e^/ others | 278 (17.9)/ 805 (51.9) / 354 (22.9) / 110 (7.1)/ 3 (0.2) | 68 (13.5)/ 306 (60.7) / 93 (18.5) / 37 (7.3)/ 0 | <0.01 |
| Days from admission to the first dose of suvorexant | 4.4 (2.7 – 7.3) | 2.3 (1.3 – 3.3) | <0.01 |
|  | Control (n=1,550) | Suvorexant (n=504) | p |
| **Non-surgical admission** | | | |
| Cardiovascular disease | 167 (10.8) | 72 (14.3) | <0.01 |
| Respiratory disease | 224 (14.4) | 54 (10.7) | 0.59 |
| Gastrointestinal disease | 86 (5.6) | 11 (2.2) | 0.02 |
| Sepsis | 66 (4.3) | 9 (1.8) | 0.06 |
| Others | 101 (6.5) | 20 (4.0) | 0.24 |
| **Subtotal** | 644 | 166 |  |
| **Surgical admission** | | | |
| Cardiovascular surgery | 704 (45.4) | 291 (57.7) | <0.01 |
| Pulmonary surgery | 43 (2.8) | 10 (2.0) | 0.17 |
| Gastrointestinal surgery | 103 (6.6) | 17 (3.3) | <0.01 |
| Others | 56 (3.6) | 20 (4.0) | 0.86 |
| **Subtotal** | 906 | 338 |  |

^a^HD:Hemodialysis

^b^CV: Cardiovascular Surgeries

^c^RRS: Rapid Response System

^d^OR: Operating Room

^e^ED: Emergency Department
