# Supplementary material for: Reducing the effect of immortal time bias affects the analysis of prevention of delirium by suvorexant in critically ill patients: A retrospective cohort study
Source: PLoS One. 2022 Dec 1;17(12):e0277916. doi: 10.1371/journal.pone.0277916 (PMC9714704; doi:10.1371/journal.pone.0277916)
Supplement: S2 Table — Other variables, co-administered medicines at the ICU, and the outcomes in “any time before” analysis. (DOCX) [file pone.0277916.s003.docx]

| Fentanyl, continuous infusion | 1,012 (65.3) | 346 (68.7) | 0.17 |
| --- | --- | --- | --- |
| Tramadol with/without acetaminophen | 163 (10.5) | 128 (25.4) | <0.01 |
| Dexmedetomidine | 154 (9.9) | 95 (18.9) | <0.01 |
| Haloperidol | 50 (3.2) | 56 (11.1) | <0.01 |
| Midazolam | 104 (6.7) | 16 (3.2) | <0.01 |
| Propofol | 281 (18.1) | 87 (17.3) | 0.66 |
| Quetiapine | 10 (0.7) | 22 (4.4) | <0.01 |
| Ramelteon | 39 (2.5) | 292 (57.9) | <0.01 |
| Steroids | 153 (9.9) | 43 (8.5) | 0.37 |
| Famotidine | 64 (4.1) | 27 (5.4) | 0.24 |
| **Outcomes** | | | |
|  | Control (n=1,550) | Suvorexant (n=504) | p |
| Delirium | 911 (58.8) | 73 (14.5) |  |
|  | HR^b^, 0.16; 95%CI^c^, 0.13 – 0.21 | | <0.01 |
| Ventilator days (hours) | 63.8 (18.0 – 163.2) | 22.2 (10.6 – 93.1) | <0.01 |
| ICU length of stay (days) | 5.9 (4.1 – 10.7) | 5.8 (4.2 – 7.9) | 0.03 |
| Hosp length of stay (days) | 26 (18 – 48) | 25 (19 – 38) | 0.60 |
| Days to the onset of delirium from the admission to the ICU (days) | 0.8 (0.4 – 1.9) | 3.9 (2.2 – 6.7) | <0.01 |
| Mortality at ICU discharge | 143 (9.2) | 14 (2.8) | <0.01 |
|  | RR^d^, 0.30; 95%CI, 0.18 – 0.52 | | <0.01 |
| Mortality at hospital discharge | 254 (16.5) | 29 (5.8) | <0.01 |
|  | RR, 0.35; 95%CI, 0.24 – ­­­0.51 | | <0.01 |

| **Other variables** | | | |
| --- | --- | --- | --- |
|  | Control (n=1,550) | Suvorexant (n=504) | p |
| High flow nasal oxygen | 422 (27.2) | 174 (34.6) | <0.01 |
| Non-invasive ventilation | 120 (7.7) | 49 (9.7) | 0.16 |
| Mechanical ventilation | 1,196 (77.2) | 387 (76.8) | 0.86 |
| Continuous renal replacement therapy | 333 (21.5) | 74 (14.7) | <0.01 |
| AKI^a^ within 24 hours from ICU admission | 141 (9.1) | 21 (4.2) | <0.01 |
| Urine output within 24 hours from ICU admission | 1131.5 (630 – 1720) | 1230 (735 – 1862.5) | 0.04 |
| **Co-administered medicines** | | | |
|  | Control (n=1,550) | Suvorexant (n=504) | p |

^a^AKI: Acute Kidney Injury

^b^HR: Hazard Ratio

^c^CI: Confidence Interval

^d^RR: Risk Ratio
