# Supplementary material for: Reducing the effect of immortal time bias affects the analysis of prevention of delirium by suvorexant in critically ill patients: A retrospective cohort study
Source: PLoS One. 2022 Dec 1;17(12):e0277916. doi: 10.1371/journal.pone.0277916 (PMC9714704; doi:10.1371/journal.pone.0277916)
Supplement: S1 File — Explanations of variables that were used to analyze in the present study. (DOCX) [file pone.0277916.s005.docx]

Variables

gender: 0 (male) 1(female)

age: years old

hight: cm

weight: kg

immunsupr: 0 (not receive immunosuppression) 1(received immunosuppression)

aids: diagnosis of AIDS, 0 (no) 1(yes)

lc: history of liver cirrhosis at the admission, 0(no) 1(yes)

hepfail: history of hepatic failure at the admission, 0(no) 1(yes)

heartfail: history of heart failure at the admission, 0(no) 1(yes)

metastases: history of metastatic cancer at the admission, 0(no) 1 (yes)

lymphoma: history of lymphoma at the admission, 0(no) 1(yes)

maintdialysis: undergoing maintenance dialysis, 0(no) 1(yes)

apache2score: APACHE 2 score

gcs_e: Glasgow Coma Scale, eye movement

gcs_m: Glasgow Coma Scale, motor response

gcs_v: Glasgow Coma Scale, verbal response

hfnc: oxygen provided by a high flow nasal canula, 0(no) 1(yes)

nppv: using a non-invasive positive pressure ventilation, 0(no) 1(yes)

vents: ventilator use during the ICU stay, 0(no) 1(yes)

irrt: intermittent renal replacement therapy, 0(no) 1(yes)

crrt: continuous renal replacement therapy, 0(no) 1(yes)

trach: undergoing tracheostomy during the ICU stay, 0(no) 1(yes)

emergecall: admission to the ICU via emergency calls, 0(no) 1(rapid response system) 2(code blue)

admcateg: admission categories, 0(non operative) 1(post elective surgeries) 2(post emergency surgeries)

readmission: not the first admission to the ICU during the index admission to the hospital, 0(no) 1(yes)

outcohos: an outcome at the hospital discharge, 0(discharged to home) 1(death) 2(discharged to other hospitals)

outcoicu: an outcome at the ICU discharge, 0(discharged to the ward) 1(death) 2(discharged to other hospitals) 3(discharged to home) 4(discharged to different unit)

urin24: amount of urine output within 24 hours from the admission, ml/24 hours

aki 24: acute kidney injury developed within 24 hours from the admission, 0(no) 1(yes)

admhospdate: the date of admission to the hospital

dischdate: the date of discharge from the hospital

admicutime: the time of the ICU admission

dischargeicutime: the time of the ICU discharge

delirium: developing delirium diagnosed by CAM-ICU, 0(no) 1(yes)

delirium_start: the time of the first delirium diagnosed by CAM-ICU

fenta500all: administration of fentanyl, 0(no) 1(yes)

tramadolall: administration of tramadol, 0(no) 1(yes)

dexall: administration of dexmedetomidine, 0(no) 1(yes)

haloperidolall: administration of haloperidol, 0(no) 1(yes)

midazolam10bin: administration of midazolam, 0(no) 1(yes)

prop500bin: administration of propofol, 0(no) 1(yes)

quetiapineall: administration of quetiapine, 0(no) 1(yes)

exposure_R: administration of ramelteon, 0(no) 1(yes)

exposure_S: administration of suvorexant within 72 hours from the admission, 0(no) 1(yes)

exposure_alt: administration of suvorexant any time before delirium development, 0(no) 1(yes)

staroidall: administration of any kind of steroid, 0(no) 1(yes)

famotidineall: administration of famotidine, 0(no) 1(yes)

admdatenum: the date of the hospital admission

dischdatenum: the date of the hospital discharge

admicunum: the time of the ICU admission

dischicunum: the time of the ICU discharge

delstartnum: the time of the delirium start

vents1begin_num: the time of the mechanical ventilation begins

vents1end_num: the time of the mechanical ventilation ends

admtodel: the time to the onset of delirium from the ICU admission, hours

vents1durat: the length of the mechanical ventilation, hours

iculos: the length of the ICU stay, hours

hosplos: the length of the hospital stay, days

bmi: body mass index

nonsurgadm: non surgical admission, 0(surgical admission) 1(cardiovascular diseases) 2(respiratory diseases) 3(gastrointestinal diseases) 4(neurological diseases) 5(sepsis) 6(traumas, non surgical) 7(metabolic diseases) 8(hematological diseases) 9(genitourinary diseases) 10(others) 11(musculoskeletal diseases)

surgadm: surgical admission, 0(non surgical admission) 12(cardiovascular surgeries) 13(pulmonary surgeries) 14(gastrointestinal surgeries) 15(neurological surgeries) 16(trauma surgeries) 17(genitourinary surgeries) 18(gynecological/obstetrical surgeries) 19(musculoskeletal surgeries) 21(surgeries for hematological diseases) 22(surgeries for metabolic organ)

daystodel: days to the onset of delirium from the admission to the ICU, days

timetosuvo: time to the administration of suvorexant from the admission to the ICU, hours

daystosuvo: days to the administration of suvorexant from the admission to the ICU, days
