# Supplementary material for: Reducing the effect of immortal time bias affects the analysis of prevention of delirium by suvorexant in critically ill patients: A retrospective cohort study
Source: PLoS One. 2022 Dec 1;17(12):e0277916. doi: 10.1371/journal.pone.0277916 (PMC9714704; doi:10.1371/journal.pone.0277916)
Supplement: Attachment — Submitted filename: PONE-D-22-2119_REVIEWER.docx [file pone.0277916.s006.docx]

**Reducing the effect of immortal time bias affects the analysis of prevention of delirium by suvorexant: A retrospective cohort study**

Comments

This is an interesting study in term of the study design to lessen the immortal time bias for the effect of suvorexant on the occurrence of delirium. Even the favorable outcomes of suvorexant on the incidence of delirium was addressed in previous studies but some limitations regarding the time of suvorexant administration was not clearly identified. I am impressed for the concept of this study; however, the retrospective cohort design has resulted in several limitation for interpretation of the outcomes. Given that, the imbalance in baseline characteristics between control and exposed groups would be the main concern.

Several comments need to be delineated.

- Title :
- I recommend to add “ in critically ill patients” at the end of the title
- Introduction:
- Please add reference for the sentence in line 67,69,76
- Line 77 “summarized” (past tense)
- Line 97, please provide the rationale for using the time frame “within 72 h” ,

any other previous studies mentioned about the onset of delirium in Japanese ICUs?

- Methods:

-If delirium had occurred several time during ICU stay, the investigator would collect only the first time?

- Although it was mentioned in the limitation, the author should give more information regarding the general indications and the dosage of suvorexant because suvorexant is not available in many countries. Any other indication than insomnia? Who have ordered? Intensivist? Psychiatrist? Geriatrician? This issue might interfere with the outcomes.

- Please provide the data regarding the accuracy of CAM-ICU (sens & spec) in practice because there were studies reported lower accuracy for CAM-ICU in practice.

- Did you have any data about the types of delirium (hypo,hyper,mixed) and other non-pharmacologic intervention (ABCDEF bundles) for prevention of delirium? Because non-pharmacologic intervention showed more effectively in prevention of delirium in ICU than pharmacologic prevention. Did you control for this intervention between groups?

- Please provide information about sample size calculation

- Results:

-There was significantly different in baseline characteristics between 2 groups in both analyses. This issue should be mentioned in limitation section. Patients in suvorexant showed higher number of surgical patients especially post cardiac surgery that seem to be less severity than control group.

-The onset of delirium was only 0.8 day in control group? Was it hypo or hyperactive delirium? If it was hypoactive delirium, it might result from the anesthesia -related effect?

- Could you explain whether ramelteon wasn’t adjusted in both analyses? Ramelteon is a melatonin receptor agonist that might improve sleep quality and decrease delirium in ICUs (even still inconclusive)

- Please provide the title of all figures

- Discussion:
- Please add detail in limitation section as mentioned above
- Limitations, consider word “first” because second, third,… were not mentioned?

Please re-check English grammar
